# Association Between the Acute to Chronic Workload Ratio and Injury Occurrence in Young Male Team Soccer Players: A Preliminary Study

**DOI:** 10.3389/fphys.2020.00608

**Published:** 2020-06-24

**Authors:** Hamid Arazi, Abbas Asadi, Farhood Khalkhali, Daniel Boullosa, Anthony C. Hackney, Urs Granacher, Hassane Zouhal

**Affiliations:** ^1^ Department of Exercise Physiology, Faculty of Sport Sciences, University of Guilan, Rasht, Iran; ^2^ Department of Physical Education and Sport Sciences, Payame Noor University, Tehran, Iran; ^3^ INISA, Federal University of Mato Grosso do Sul, Campo Grande, Brazil; ^4^ Sport and Exercise Science, James Cook University, Townsville, QLD, Australia; ^5^ Department of Exercise and Sport Science, University of North Carolina, Chapel Hill, NC, United States; ^6^ Division of Training and Movement Sciences, University of Potsdam, Potsdam, Germany; ^7^ M2S (Laboratoire Mouvement, Sport, Santé)-EA 1274, Univ Rennes, Rennes, France

**Keywords:** training load, rate of perceived exertion, rolling averages, weighted moving averages, football

## Abstract

This study aimed to investigate the relationship between the acute to chronic workload ratio (ACWR), based upon participant session rating of perceived exertion (sRPE), using two models [(1) rolling averages (ACWR_RA_); and (2) exponentially weighted moving averages (ACWR_EWMA_)] and the injury rate in young male team soccer players aged 17.1 ± 0.7 years during a competitive mesocycle. Twenty-two players were enrolled in this study and performed four training sessions per week with 2 days of recovery and 1 match day per week. During each training session and each weekly match, training time and sRPE were recorded. In addition, training impulse (TRIMP), monotony, and strain were subsequently calculated. The rate of injury was recorded for each soccer player over a period of 4 weeks (i.e., 28 days) using a daily questionnaire. The results showed that over the course of the study, the number of non-contact injuries was significantly higher than that for contact injuries (2.5 vs. 0.5, *p* = 0.01). There were also significant positive correlations between sRPE and training time (*r* = 0.411, *p* = 0.039), ACWR_RA_ (*r* = 0.47, *p* = 0.049), and ACWR_EWMA_ (*r* = 0.51, *p* = 0.038). In addition, small-to-medium correlations were detected between ACWR and non-contact injury occurrence (ACWR_RA_, *r* = 0.31, *p* = 0.05; ACWR_EWMA_, *r* = 0.53, *p* = 0.03). Explained variance (*r* ^2^) for non-contact injury was significantly greater using the ACWR_EWMA_ model (ranging between 21 and 52%) compared with ACWR_RA_ (ranging between 17 and 39%). In conclusion, the results of this study showed that the ACWR_EWMA_ model is more sensitive than ACWR_RA_ to identify non-contact injury occurrence in male team soccer players during a short period in the competitive season.

## Introduction

In contemporary soccer, it is important to understand the sport-specific physiological demands for performance development and injury prevention as the game has become much faster and demanding over the past two decades (e.g., soccer players typically run ~11 km per game with 30–40 short sprints, ~600 accelerations, ~25 high-intensity accelerations, ~600 decelerations, ~45 high-intensity decelerations, and more than 1,300 change of direction activities) (Barnes et al., 2014; Andrzejewski et al., 2015; Russell et al., 2016). The importance of adequate training regimes during pre- and in-season is required so that athletes are well-prepared for the season. This is particularly important in youth soccer given that growth and maturation in general and the individual timing and tempo of these two factors additionally predispose players to injury. There is evidence that injury rates are particularly high during puberty (Fort-Vanmeerhaeghe et al., 2016).

To be prepared for these requirements, it is recommended that soccer players train several times during the week and, to increase the chances of success, coaches implement specific training loads to challenge the boundaries of what players can achieve without exceeding what their bodies can tolerate (Ehrmann et al., 2016; Bowen et al., 2017). However, an appropriate balance between training, competition, and recovery is required to reach peak performances with minimal injury rates, but this is an elusive goal. Thus, understanding and monitoring training programs of soccer players are crucial to ensure that an optimal training load is applied (Gabbett and Ullah, 2012).

It is recommended to monitor young soccer players during training because an adequate load is essential for short-term performance development as well as for enabling the future potential of these athletes (Ehrmann et al., 2016). In fact, 30–50% of injuries are estimated to result from overuse during training and therefore, it is important to emphasize the correct monitoring of training variables (i.e., load) to optimize growth, development, and fundamental movement skills by reducing injury risk and rate (Fort-Vanmeerhaeghe et al., 2016).

The scientific literature provides different methods, which have previously established associations between measures of training load and injury rate in athletes. In many studies, the acute to chronic workload ratio (ACWR) was used (Hulin et al., 2015; Soligard et al., 2016; Enright et al., 2020; Griffin et al., 2020; Myers et al., 2020). The ACWR is an index of athletes’ training stress that evolved in relation to the fitness level in response to a training session as accrued through their chronic exposure to training (Gabbe et al., 2006). Even though critical reports exist on the ACWR (Hulin et al., 2015), the International Olympic Committee (IOC) has recommended using the ACWR to monitor injury and to provide athletes’ thresholds to minimize injury occurrence throughout training programs (Soligard et al., 2016).

The ACWR can be calculated using two models, the rolling average model (ACWR_RA_) and the exponentially weighted moving average model (ACWR_EWMA_). The ACWR_RA_ model is calculated by dividing the current workload (i.e., acute, 7-day workload) with respect to the workload that an athlete has completed for the current preparedness (i.e., chronic, 28-day workload). In contrast, the ACWR_EWMA_ model, which was first presented by Williams et al. (2017), determines ACWR by assigning a decreasing weighting for each older load value in order to give greater weighting to the recent load performed by the athlete.

The use of ACWR is based on the fitness-fatigue theory of the body’s response to training (Gabbe et al., 2006), and was developed to assist practitioners to better manage the preparation of athletes for competition while considering the risk of overtraining and injury (Hulin et al., 2015). The use of the ACWR has received a growing interest in recent years to monitor loads during competition and training and to determine injury in a variety of team sports. For this purpose, internal session rating of perceived exertion (sRPE) and external measures (tracking variables; i.e., GPS, time) are used (Blanch and Gabbett, 2016; Enright et al., 2020; Griffin et al., 2020; Myers et al., 2020).

To date, various studies explored the relationship between training load (i.e., acute and chronic) and injury occurrence in athletes using both ACWR models. Previously, Anderson et al. (2003) reported a moderate correlation between total training load and incidence of injury, within 1 week of training, in division III female basketball players using ACWR_RA_. In elite athletes, a number of studies previously explored potential associations between ACWR and injury rate. Findings from these studies are controversial in as much as some researchers found positive associations between ACWR and injury rate in sports, such as soccer (Bowen et al., 2017), American football (Carey et al., 2017; Colby et al., 2017; Murray et al., 2017), and rugby (Cross et al., 2016) and consequently postulated that the monitoring of workloads provides valuable information for injury occurrence, while other researchers were more critical because their data did not show any value of the ACWR to predict injury rate in athletes (Fanchini et al., 2018; Impellizzeri et al., 2020).

While previous studies aimed to clarify the relationship between training loads and injury rate in adult athletes, to date, there is limited research (Bowen et al., 2017; Delecroix et al., 2018) exploring the validity of ACWR monitoring for injury rate in youth soccer players during a competitive phase using both the ACWR_EWMA_ and ACWR_RA_ models. Given the controversial findings in the literature with some studies reporting positive (Cross et al., 2016; Bowen et al., 2017; Carey et al., 2017; Colby et al., 2017; Murray et al., 2017; Griffin et al., 2020; Myers et al., 2020) while others negative associations between different ACWR models and injury rate in athletes (Fanchini et al., 2018; Impellizzeri et al., 2020), the aim of this study was to examine the relationship between two ACWR models (i.e., ACWR_RA_ and ACWR_EWMA_) and injury rate in young team soccer players during a short competitive period. With reference to the relevant literature (Foster, 1998; Enright et al., 2020), we hypothesized that particularly ACWR_EWMA_ could be related with injury rate during a competitive mesocycle.

## Materials and Methods

### Participants

Data were collected from a semi-professional U-18 soccer team (*N* = 22, age: 17.1 ± 0.7 years, height: 175.4 ± 5.6 cm, body mass: 76.3 ± 6.4 kg). The team played competitive fixtures in the Second Division of the Iranian National League during the 2019 season. The soccer team included 25 players. Three of them were goalkeepers. Due to the different demands of their activities, the goalkeepers were excluded from this study. The remaining field players consisted of eight defenders (36.3%, two right full backs, two left full backs, and four center backs), nine midfielders (40.9%, four wingers, two defending midfielders, and three central midfielders), and five forwards (22.7%, three forwards, and two strikers). The sample size was calculated based on a previous study by Foster (1998) with an alpha level of 0.05, and an actual power (1-beta) of 0.80. *A priori* power analysis was computed using G × Power (Version 3.1.9.2, University of Kiel, Germany) and the *t*-test family. The analysis revealed that a total sample size of *N* = 21 would be sufficient to find significant and medium-sized correlations between workload and injury rate.

The players exercised according to their playing position throughout the study period. The training program was designed and prescribed by the team coaches. Each training session included a short warm up program, technical and tactical drills as well as a strength and conditioning program including linear sprints and plyometrics. All soccer players exercised at the same time according to the demands of their playing position (Table 1).

**Table 1:** Training program over the course of the observation period.

	Monday	Tuesday	Wednesday	Thursday	Friday	Saturday	Sunday
Morning session	Recovery	Individual fitness Technical-tactical training	Technical-tactical training	Individual fitness Technical-tactical training	Individual fitness Technical-tactical training	Individual fitness Technical-tactical training	Official game
Afternoon session	Recovery	Video or multidisciplinary activities	Individual fitness^*^	Specific training	Recovery	Recovery	Recovery

Here, we exemplified 1 week of training.

* Individual fitness: includes strength and conditioning exercises which were applied in accordance with the players’ position on the pitch.

Ethical approval was obtained from the research ethics committee of the University of Guilan, Iran. The study was conducted in accordance with the latest version of the Declaration of Helsinki.

### ACWR Calculation

The ACWR of internal training load (i.e., sRPE) was calculated using two models including the rolling average (ACWR_RA_) and the exponentially weighted moving average (ACWR_EWMA_). The ACWR_RA_ was calculated by dividing the acute workload (1-week rolling workload data) by the chronic workload (the rolling 4-week average workload data) (Hulin et al., 2015; Foster et al., 2018; Myers et al., 2020).

The ACWR_EWMA_ was calculated as: EWMA^today^ = Load^today^ × ƛ_a_ + [(1-ƛ) × EWMA^yesterday^]. In this formula ƛ_a_ is calculate by 2/(N + 1) ranging value between 0 and 1 that represents a decay rate to the load value (Murray et al., 2017; Williams et al., 2017). The *N* value is the chosen time decay constant including acute (1-week) and chronic (4-weeks) periods.

These approaches were used in the previous studies which applied ACWR models to clarify associations between training load and injury rate in team sports (Hulin et al., 2015; Soligard et al., 2016; Enright et al., 2020; Griffin et al., 2020; Myers et al., 2020).

### Session Rating of Perceived Exertion

During the familiarization session, each player received instructions on how to use a modified Borg 10-point sRPE scale (Foster et al., 2001). For this purpose, standard instructions and anchoring procedures were explained (Asadi, 2014). A rating of 0 was associated with no exertion (rest) at all, and a rating of 10 was considered to be maximal exertion and associated with the most demanding exercise performed. Ten to twenty minutes following training sessions and games, players were asked “*How was your perceived exertion in this session/game*?” Players verbally indicated a number to rate their overall effort according to 0 = rest, 1 = very, very rest, 2 = easy, 3 = moderate, 4 = somewhat hard, 5 and 6 = hard, 7, 8, and 9 = very hard, and 10 = maximal. In addition, sRPE × training time in minutes was assessed for the definition of training impulse (TRIMP) (Foster et al., 2001).

### Definition of Injury Occurrence

The medical staff of the soccer club classified all injuries (i.e., contact and non-contact), with injury reports recorded and updated on a daily basis throughout the study period. Injuries were categorized by injury type (non-contact vs. contact) and body site (injury location). Injuries were classified as follows: minimal (1–3 days of soccer activity missed), mild (4–7 days of soccer activity missed), moderate (1–4 weeks of soccer activity missed), and severe (4+ weeks of soccer activity missed) depending on the days of missed activities (Rogalski et al., 2013).

### Statistical Analyses

Normality of data was tested using the Shapiro-Wilk’s test. If normality existed, we presented the data as mean and standard deviations (SDs). If not the data were presented as median and interquartile ranges (IQR). Due to the limited number of monitored injuries, the more conservative Spearman’s rank correlation coefficient was used to determine the relationships between training monitoring tools (i.e., ACWR_RA_ and ACWR_EWMA_) and injury occurrence. The magnitude of the correlations was considered 0.1–0.29, small; 0.30–0.49, medium; and >0.50, large (Chen and Popovich, 2002). A Kernel regression analysis was performed to determine the variance (*r* ^2^) in injury occurrence (i.e., non-contact injury) explained by the TRIMP, sRPE, training time, strain, ACWR_RA_, and ACWR_EWMA_. All analyses were performed using SPSS statistics (IBM SPSS Software, v21.0, Armonk, NY, USA). The level of significance was set at *p* ≤ 0.05. The TRIMP was computed by multiplying the duration of each training session with the respective session RPE. This was computed for each player individually. The daily mean divided by the respective SD was calculated and used as an expression of monotony. The product of the weekly training load and monotony was calculated as strain. The weekly load was determined by multiplying the daily mean load by 7 (Foster, 1998).

## Results

**Figure 1:**
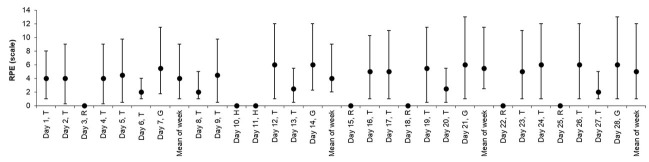
The session rating of perceived exertion (sRPE) over the course of the study period and the mean for each week. Data are presented as median and interquartile ranges (median-IQR). T, training session; R, rest; G, game; H, holiday.

**Figure 2:**
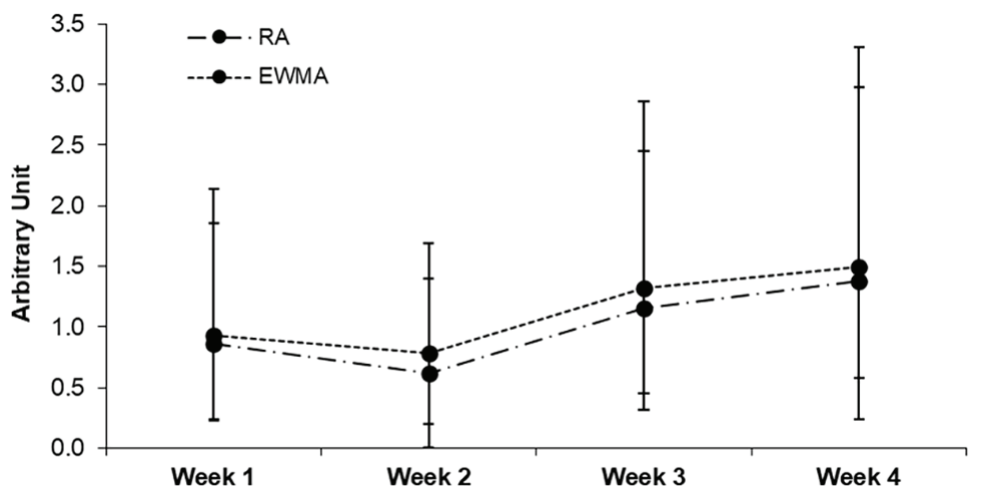
Acute to chronic workload ratio (ACWR). Data are presented as median-IQR. RA, rolling average; EWMA, exponentially weighted moving average.

Table 2 presents descriptive data for sRPE, training time, TRIMP, monotony, weekly load, and strain for both ACWR models. Figures 1, 2 contain data across the 28 days observation period for sRPE and ACWR, respectively. Table 3 provides information on the number of injuries for contact and non-contact injuries experienced during the study period. The results showed a significantly larger number of non-contact compared with contact injuries (*p* = 0.01).

**Table 2:** Workload parameters assessed and computed for this study.

	sRPE (scale)	Training time (min)	TRIMP^*^	Monotony*^†^*	Weekly load*^‡^*	Strain*^€^*	Number of injuries
Week 1	4.0(1–6)	75(60–90)	300(150–400)	3.3	2,100	6,930	3
Week 2	4.1(2–5)	90(60–90)	210(120–380)	2.3	1,470	3,381	3
Week 3	5.5(2–7)	80(60–90)	400(100–450)	3.0	2,800	8,400	4
Week 4	5.0(2–7)	85(60–100)	480(120–540)	5.0	3,360	16,800	5

Data are presented as median-IQR. sRPE, sessions rating of perceived exertion.

* TRIMP = RPE × training time.

† Monotony = daily mean load/standard deviation (SD).

‡ Weekly load = daily mean load × 7.

€ Strain = weekly load × monotony (Foster, 1998).

**Table 3:** Classification of the identified injuries over the course of the study.

Site	Non-contact	Contact	Injury type	Severity
Ankle/foot	2	1	Sprain	Minimal
Knee	3	2	Joint injury	Mild
Hip	4	0	Strain	Minimal
Quadriceps	2	1	Strain	Minimal
Hamstring	1	0	Strain	Minimal
Gastrocnemius	3	0	Strain	Minimal
Median (IQR)	2.5(1.75–3.25)^*^	0.5(0–1.25)		

* Significant differences compared to contact injury (*p* = 0.01).

The analysis revealed a positive medium-sized correlation between sRPE and training time (*r* = 0.411, *p* = 0.039). In addition, medium- and large-sized correlations were found between weekly sRPE and ACWR_RA_ (*r* = 0.47, *p* = 0.049) as well as ACWR_EWMA_ (*r* = 0.51, *p* = 0.038). Likewise, medium-and large-sized correlations were found between the ACWR and injury occurrence during the competitive mesocycle (ACWR_RA_, *r* = 0.31, and *p* = 0.050; ACWR_EWMA_, *r* = 0.53, and *p* = 0.03).

For ACWR_RA_, our findings indicated 17% (*r* ^2^ = 0.17, *p* = 0.7), 34% (*r* ^2^ = 0.34, *p* = 0.05), 39% (*r* ^2^ = 0.39, *p* = 0.048), and 27% (*r* ^2^ = 0.27, *p* = 0.56) of the explained variance for the sRPE, training time, TRIMP, and strain, respectively. For ACWR_EWMA_, 21% (*r* ^2^ = 0.21, *p* = 0.052), 40% (*r* ^2^ = 0.40, *p* = 0.039), 52% (*r* ^2^ = 0.52, *p* = 0.018), and 28% (*r* ^2^ = 0.28, *p* = 0.05) of explained variance were found for sRPE, training time, TRIMP, and strain, respectively.

## Discussion

The aim of this preliminary study was to investigate the relationships between internal workload parameters and injury occurrence in young team soccer players throughout a 4-weeks competitive mesocycle. We found that the sRPE increased from day 1 to 28 and, that the mean sRPE increased from week 1 to 4, which indicates a progressive increase in training load for all players. In addition, TRIMP increased from week 1 to 4 following the scheduled periodized training program of the team coaches. Moreover, the current data revealed medium- to large-sized correlations between training time (min/week) and ACWR (both the ACWR_RA_ and ACWR_EWMA_) with sRPE, which indicates positive associations between training volume and sRPE, and between load periodization and sRPE in athletes. Further, we found significant medium- to large-sized relationships between measures of internal load and the number of non-contact injuries.

This study revealed that weekly sRPE is significantly and positively correlated with the ACWR score. This is in line with Foster et al. (2018) who reported positive large-sized correlations between the ACWR (both the ACWR_RA_ and ACWR_EWMA_ models) and weekly sRPE (*r* = 0.6, *p* < 0.01) in females over a 4-week training period. In addition, Myers et al. (2020) examined junior tennis players and observed positive relationships between the ACWR_RA_ and sRPE across the training period. Taking our findings together with those reported in the literature, it seems possible to postulate that sRPE is an appropriate marker to monitor training load.

The most important finding of this study was the medium- to large-sized relationship between ACWR and the number of non-contact injuries in young soccer players. Our results revealed that this association was larger for the ACWR_EWMA_ compared with the ACWR_RA_ (*r* = 0.53 vs. 0.31). In line with our findings, Hulin et al. (2015), Bowen et al. (2017), and Murray et al. (2017) have previously shown significant and large-sized relationships between the ACWR and injury rate in elite male adult soccer and football players. In addition, Griffin et al. (2020) found that the ACWR_EWMA_ model is a more sensitive measure of ACWR in comparison to the ACWR_RA_ model of team athletes. In contrast, Fanchini et al. (2018) and Impellizzeri et al. (2020) have reported that the monitoring of training loads using the ACWR has no scientific merit to predict injury risk and rate of athletes, which is why they recommended not to use ACWR methods in the context of injury prevention (Enright et al., 2020). Given the described discrepancy in the scientific literature, there is a general need to conduct more research on the ACWR. More specifically, the potential relationships of different ACWR models with injury risk and rate should continue to be scrutinized in future studies.

To date, it appears that ACWR <1 is associated with a lower injury occurrence in athletes and ACWR >1.5 appear to be associated with an increased injury rate (Hulin et al., 2015). In agreement with these findings, Malone et al. (2017) showed that increased weekly workloads resulted in an increased injury rate in professional soccer players. On the other hand, there were positive large associations between ACWR and injury occurrence in athletes, which indicated increased training load, induced greater injury rate in athletes (Hulin et al., 2015; Myers et al., 2020). Recently, Griffin et al. (2020) in a systematic review article explained the observed associations between the ACWR and injury in team sports. Findings of this study supported the association between the ACWR and non-contact injuries and more sensitive of EWMA model for monitoring training load as part of a larger scale multifaceted monitoring system. In addition, the majority of studies reported non-contact soft-tissue injuries following increases in ACWR (Gabbett, 2010).

Within the current literature, it has been suggested that when the chronic workload is much greater than the acute workload, it results in a low ACWR; i.e., a lower rate of injury can be observed (Murray et al., 2017). Conversely, increases in the ACWR result in a higher rate of injury (Bowen et al., 2017). Although, both the ACWR models demonstrated significant relationships with injury occurrence, there were notable differences between the ACWR_RA_ and ACWR_EWMA_ models to injury occurrence. In this study, the ACWR_EWMA_ explained between 21 and 52% in variance (*r* ^2^) for the sRPE, training time, TRIMP, and strain, while the variance explained by the ACWR_RA_ was minimally significant lower (between 17 and 39%). In line with these findings, Murray et al. (2017) and Griffin et al. (2020) reported that in-between ACWR models, the EWMA is a more sensitive measure than RA in injury occurrence of team athletes. With the continued advancement and use of modern monitoring technology, these findings provide useful information for strength and conditioning staff of young soccer teams to monitor the ACWR and its relationship with an individual player’s workloads and their injury rates.

### Limitations

This preliminary study has a few methodological limitations that warrant discussion. First, the number of included athletes (i.e., 22 soccer players) was rather low, affecting the power of the study. However, we conducted the priori power analysis and showed that this number appears to be adequate to receive sufficient statistical power. Second, the observation period was rather short. Yet, other researchers have previously reported that 28 days (7:28 ratio) are sufficient to calculate RA and EWMA and to determine the relationship between workload variables and injury occurrence in soccer players (Delecroix et al., 2018; Griffin et al., 2020). Third, findings from this study are specific to U-18 male soccer players. More research is needed to identify whether our findings can be transferred to females and soccer players of different age categories. Finally, it would have been interesting to include other variables as an estimate for external training load such as GPS data. This should be done in future studies to verify our results. Taking these limitations together, we consider our findings preliminary and thus, there is a need for future work to refute or support our outcomes.

## Conclusion

In conclusion, findings from this preliminary study demonstrated that the ACWR using sRPE and training time is easy-to-administer and useful as a measure to monitor injury occurrence in U-18 male soccer. The most important result of this study is that ACWRs calculated with exponential weighting have stronger correlations (associations) to injury occurrence than rolling averages in male team soccer players.

## Data Availability Statement

The raw data supporting the conclusions of this article will be made available by the authors, without undue reservation.

## Ethics Statement

The studies involving human participants were reviewed and approved by Ethical approval were obtained from the research ethics committee of the University of Guilan, Iran. The study was conducted in accordance with the latest version of the Declaration of Helsinki. Written informed consent to participate in this study was provided by the participants’ legal guardian/next of kin.

## Author Contributions

HA, AA, and HZ designed the study. FK collected the data. HA and AA conducted the analyses. HA, AA, HZ, and FK wrote the manuscript. HA, HZ, UG, DB, and AH were involved in the interpretation of data, and reviewed and edited the manuscript. HZ and UG made a substantial and intellectual contribution to the conception and a critical revision of the manuscript. All authors read and approved the final version of the manuscript.

## Conflict of Interest

The authors declare that the research was conducted in the absence of any commercial or financial relationships that could be construed as a potential conflict of interest.

## References

[ref1] Anderson L. Triplett-McBride T. Foster C. Doberstein S. Brice G. (2003). Impact of training patterns on incidence of illness and injury during a women’s collegiate basketball season. J. Strength Cond. Res. 17, 734–738. 10.1519/1533-4287(2003)017<0734:iotpoi>2.0.co;2, PMID: 14636112

[ref2] Andrzejewski M. Chmura J. Pluta B. (2015). Sprinting activities and distance covered by top level Europa league soccer players. Int. J. Sports Sci. Coach. 10, 39–50. 10.1260/1747-9541.10.1.39

[ref3] Asadi A. (2014). Use of rating of perceived exertion for determining plyometric exercises intensity in physically active men. Sports Sci. Health 10, 75–78. 10.1007/s11332-014-0176-y

[ref4] Barnes C. Archer D. T. Hogg B. Bush M. Bradley P. S. (2014). The evolution of physical and technical performance parameters in the English premier league. Int. J. Sports Med. 35, 1095–1100. 10.1055/s-0034-1375695, PMID: 25009969

[ref5] Blanch P. Gabbett T. J. (2016). Has the athlete trained enough to return to play safely? The acute:chronic workload ratio permits clinicians to quantify a player’s risk of subsequent injury. Br. J. Sports Med. 50, 471–475. 10.1136/bjsports-2015-095445, PMID: 26701923

[ref6] Bowen L. Gross A. S. Gimpel M. Li F. X. (2017). Accumulated workloads and the acute: chronic workload ratio relate to injury risk in elite youth football players. Br. J. Sports Med. 51, 452–429. 10.1136/bjsports-2015-095820, PMID: 27450360 PMC5460663

[ref7] Carey D. L. Blanch P. Ong K. L. Crossley K. M. Crow J. Morris M. E. (2017). Training loads and injury risk in Australian football-differing acute: chronic workload ratios influence match injury risk. Br. J. Sports Med. 51, 1215–1220. 10.1136/bjsports-2016-096309, PMID: 27789430 PMC5537557

[ref8] Chen P. Y. Popovich P. M. (2002). Correlation: Parametric and nonparametric measures. Thousand Oaks, CA: Sage Publications.

[ref9] Colby M. J. Dawson B. Peeling P. Heasman J. Rogalski B. Drew M. K. (2017). Multivariate modelling of subjective and objective monitoring data improve the detection of non-contact injury risk in elite Australian footballers. J. Sci. Med. Sport 20, 1068–1074. 10.1016/j.jsams.2017.05.010, PMID: 28595869

[ref10] Cross M. J. Williams S. Trewartha G. Kemp S. P. Stokes K. A. (2016). The influence of in-season training loads on injury risk in professional rugby union. Int. J. Sports Physiol. Perform. 11, 350–355. 10.1123/ijspp.2015-0187, PMID: 26309331

[ref11] Delecroix B. McCall A. Dawson B. Berthoin S. Dupont G. (2018). Workload and non-contact injury incidence in elite football players competing in European leagues. Eur. J. Sport Sci. 18, 1280–1287. 10.1080/17461391.2018.1477994, PMID: 29860935

[ref12] Ehrmann F. E. Duncan C. S. Sindhusake D. Franzsen W. N. Greene D. A. (2016). GPS and injury prevention in professional soccer. J. Strength Cond. Res. 30, 360–367. 10.1519/JSC.0000000000001093, PMID: 26200191

[ref13] Enright K. Green M. Hay G. Malone J. J. (2020). Workload and injury in professional soccer players: role of injury tissue type and injury severity. Int. J. Sports Med. 41, 89–97. 10.1055/a-0997-6741, PMID: 31801172

[ref14] Fanchini M. Rampinini E. Riggio M. Coutts A. Pecci C. McCall A. (2018). Despite association, the acute:chronic work load ratio does not predict non-contact injury in elite footballers. Sci. Med. Football 2, 108–114. 10.1080/24733938.2018.1429014

[ref15] Fort-Vanmeerhaeghe A. Romero-Rodriguez D. Montalvo A. M. Kiefer A. W. Lloyd R. S. Myer G. D. (2016). Integrative neuromuscular training and injury prevention in youth athletes. Part I: identifying risk factors. Strength Cond. J. 38, 36–48. 10.1519/SSC.0000000000000229

[ref16] Foster C. (1998). Monitoring training in athletes with reference to overtraining syndrome. Med. Sci. Sports Exerc. 30, 1164–1168. 10.1097/00005768-199807000-00023, PMID: 9662690

[ref17] Foster I. Byrne P. J. Moody J. A. Fitzpatrick P. A. (2018). Monitoring training load using the acute: chronic workload ration in non-elite intercollegiate female athletes. ARC J. Res. Sports Med. 3, 22–28.

[ref100] Foster C. Florhaug J. A. Franklin J. Gottschall L. Hrovatin L. A. Parker S. (2001). A new approach to monitoring exercise training. J. Strength Cond. Res. 15, 109–115. 11708692

[ref18] Gabbe B. J. Bennell K. L. Finch C. F. Wajswelner H. Orchardm J. W. (2006). Predictors of hamstring injury at the elite level of Australian football. Scand. J. Med. Sci. Sports 16, 7–13. 10.1111/j.1600-0838.2005.00441.x, PMID: 16430675

[ref19] Gabbett T. J. (2010). The development and application of an injury prediction model for noncontact, soft-tissue injuries in elite collision sport athletes. J. Strength Cond. Res. 24, 2593–2603. 10.1519/JSC.0b013e3181f19da4, PMID: 20847703

[ref20] Gabbett T. J. Ullah S. (2012). Relationship between running loads and soft tissue injury in elite team sport athletes. J. Strength Cond. Res. 26, 953–960. 10.1519/JSC.0b013e3182302023, PMID: 22323001

[ref21] Griffin A. Kenny I. C. Comyns T. M. Lyons M. (2020). The association between the acute:chronic workload ratio and injury and its application in team sports: a systematic review. Sports Med. 50, 561–580. 10.1007/s40279-019-01218-2, PMID: 31691167

[ref22] Hulin B. T. Gabbett T. J. Blanch P. Chapman P. Bailey D. Orchard J. W. (2015). Spikes in acute workload are associated with increased injury risk in elite cricket fast bowlers. Br. J. Sports Med. 48, 708–712. 10.1136/bjsports-2013-092524 23962877

[ref23] Impellizzeri F. M. Woodcock S. Coutts A. J. Fanchini M. McCall A. Vigotsky A. D. (2020). Acute to random chronic workload ratio is ‘as’ associated with injury as acute to actual chronic workload ratio: time to dismiss ACWR and its components. *Society for Transparency. Openness and Replication in Kinesiology*. SportRxiv [Preprint]. 10.31236/osf.io/e8kt4

[ref24] Malone S. Owen A. Newton M. Mendes B. Collins K. D. Gabbett T. J. (2017). The acute:chonic workload ratio in relation to injury risk in professional soccer. J. Sci. Med. Sport 20, 561–565. 10.1016/j.jsams.2016.10.014, PMID: 27856198

[ref25] Murray N. B. Gabbett T. J. Townshend A. D. Blanch P. (2017). Calculating acute:chronic workload ratios using exponentially weighted moving averages provides a more sensitive indicator of injury likelihood than rolling averages. Br. J. Sports Med. 51, 749–754. 10.1136/bjsports-2016-097152, PMID: 28003238

[ref26] Myers N. L. Aguilar K. A. Mexicano C. Farnsworth J. L. Knudson D. Kibler W. B. (2020). The acute: chronic workload ratio is associated with injury in junior tennis players. Med. Sci. Sports Exerc. 52, 1196–1200. 10.1249/MSS.0000000000002215, PMID: 31764467

[ref27] Rogalski B. Dawson B. Heasman J. Gabbett T. J. (2013). Training and game loads and injury risk in elite Australian footballers. J. Sci. Med. Sport 16, 499–503. 10.1016/j.jsams.2012.12.004, PMID: 23333045

[ref28] Russell M. Sparkes W. Northeast J. Cook C. J. Love T. D. Bracken R. M.. (2016). Changes in acceleration and deceleration capacity throughout professional soccer matchplay. J. Strength Cond. Res. 30, 2839–2844. 10.1519/JSC.0000000000000805, PMID: 25474342

[ref29] Soligard T. Schwellnus M. Alonso J. M. Bahr R. Clarsen B. Dijkstra H. P. (2016). How much is too much? (part 1) International Olympic Committee consensus statement on load in sport and risk of injury. Br. J. Sports Med. 50, 1030–1041. 10.1136/bjsports-2016-096581, PMID: 27535989

[ref30] Williams S. West S. Cross M. J. (2017). Better way to determine the acute:chronic workload ratio? Br. J. Sports Med. 51, 209–210. 10.1136/bjsports-2016-096589, PMID: 27650255

